# Supplementary material for: Climatic Zone and Soil Properties Determine the Biodiversity of the Soil Bacterial Communities Associated to Native Plants from Desert Areas of North-Central Algeria
Source: Microorganisms. 2021 Jun 23;9(7):1359. doi: 10.3390/microorganisms9071359 (PMC8303931; doi:10.3390/microorganisms9071359)
Supplement: Supplementary file 1 [file microorganisms-09-01359-s001.zip › microorganisms-1239718-supplementary.pdf]

Climatic zone and soil properties determine the biodiversity of the soil microbial communities associated to native plants from desert areas of North-Central Algeria

Bona E.[1], Massa N.[2*], Toumatia O.[3,4], Novello G.[2], Cesaro P.[2], Todeschini V.[1], Boatti L.[2,5], Mignone F.[2,5], Titouah H.[4], Zitouni A.[4], Lingua G.[2], Vuolo F.[6], Gamalero E.[2]

[1] Università del Piemonte Orientale, Dipartimento di Scienze e Innovazione Tecnologica, Piazza San Eusebio 5, 13100 Vercelli, Italy

[2] Università del Piemonte Orientale, Dipartimento di Scienze e Innovazione Tecnologica, Viale T. Michel 11, Alessandria 15121, Italy

[3] Agro-Pastoralism Research Center (APRC) Djelfa, Algeria.

[4] Laboratoire de Biologie des Systèmes Microbiens (LBSM), Ecole Normale Supérieure (ENS) de Kouba, Algiers, Algeria.

[5] SmartSeq s.r.l., spin-off of the Università del Piemonte Orientale, Viale T. Michel 11, Alessandria 15121, Italy

[6] Sacco s.r.l., Via Alessandro Manzoni 29/A, 22071, Cadorago (CO) Italy

***Corresponding author:**

Nadia Massa Ph.D.

Dipartimento di Scienze e Innovazione Tecnologica, Università del Piemonte Orientale,

Viale T. Michel 11, Alessandria 15121, Italy

Tel: +39 0131 360 231

Fax: +39 0131 360 243

E-mail: nadia.massa@uniupo.it

Table S1. Information regarding the 14 Algerian autochthonous plant species.

Table S2. Physical-chemical properties of the soils sampled from the rhizosphere of 14 different plant species in North-Central Algeria.

Table S3. Linear discriminant analysis Effect Size (LEfSe) at species level according to sampling site. All the possible comparisons are reported in the table. Light gray indicates species less present in the first sampling site than in the second one and red indicates a presence less than 50%. Dark gray indicates species more present in the first sampling site than in the second one and green indicates a presence more than 200%.

Figure S1. A) Precipitation (mm), B) Wind speed (ms^{-1}), C) Average T (°C) in the year 2018 in the six Algerian sampling sites and Alessandria town in Italy as reference.

Figure S2. A) Minimum temperature (°C), B) Maximum temperature (°C) Temperature difference (°C) in the year 2018 in the six Algerian sampling sites and Alessandria town in Italy as reference.

Figure S3. Phylum abundance (%) in A) plants, B) sampling sites and C) climatic zone.

Figure S4. CORE analysis at phylum level according to sampling site (climatic zone). The Microbiome CORE analysis identifies core taxa that remain unchanged in their composition across the whole microbial community. Two parameters are considered: sample prevalence and relative abundance (%) of a taxa. Core microbiome analysis is adopted from the core function in R package microbiome. The result of this analysis is represented in the form of heatmap of core taxa where Y-axis represent the prevalence level of core features across the detection threshold (Relative abundance) range on X-axis. Two soil sampling sites in the arid region of Ghardaïa (Algeria): Metlili and Beni Isguen. Four soil sampling sites in the semi-arid region of Djelfa (Algeria): Messaad, Ain Naga, Moudjbara and Zaafrane.

Figure S5. CORE analysis at species level according to sampling site.

Figure S6. CORE analysis at phylum level according to climatic zone.

Figure S7. CORE analysis at species level according to climatic zone.

Figure S8. Heat trees at phylum level. Heat trees report the effect of the sampling site on hierarchical structure of taxonomic classifications (median abundance, non-parameter Wilcoxon Rank Sum test).The reported comparisons are **Metlili** *vs* A) Ain Naga, B) Beni Isguen, C) Messaad, D) Moudjbara, E) Zaafrane; **Beni Isguen** *vs* F) Ain Naga, G) Messaad, H) Moudjbara, I) Zaafrane; **Messaad** *vs* J) Ain Naga, K) Moudjbara, L) Zaafrane; **Ain Naga** *vs* M) Moudjbara, N) Zaafrane and finally **Moudjbara** *vs* O) Zaafrane. Comparing Sample 1 *vs* Sample 2, a blue line indicates that one phylum in sample 2 is more abundant than in sample 1, while a red line indicates that one phylum in sample 2 is less abundant than in sample 1. Heat tree analysis was performed using R metacoder package of MicrobiomeAnalyst, a free available on-line software (https://www.microbiomeanalyst.ca).

Table S1. Information regarding the 14 Algerian autochthonous plant species.

Label	Sampling site	Coordinates	Species	Local name	Family	Traditional uses
1	Metlili, Ghardaïa	32°16'49.9"N 3°38'17.1"E	*Cleome arabica*	Khanza (stinker), Dafrah (ذفرة عربية)	Capparaceae	It is used in the treatment of inflammation and rheumatism. It possesses antimicrobial and antioxidant activities [19]. It is also used as diuretic and febrifuge. Furthermore, *Cleome arabica* has cytotoxic effects which are exploited for the production of bioherbicides and larvicides [19,20].
2	Metlili, Ghardaïa	32°16'48.3"N 3°38'15.9"E	*Reseda villosa*	Bliha (بليحاء)	Resedaceae	It is used in medicine for its content in flavonoids with antioxidant power [21].<br>It is used in the treatment of diarrhea and intestinal pains [22].
3	Beni Isguen, Ghardaïa	32°27'52.0"N 3°41'02.7"E	*Zilla spinosa*	Shibrim (شبرم)	Brassicaceae	It is used for grazing [23] and for medicinal purposes as gastrointestinal disorders, diabetes, urinary tract pains, diarrhea, gall bladder, kidney stones, liver and pancreas pain, respiratory ailments and rheumatism [24].
4	Beni Isguen dam, Ghardaïa	32°27'54.1"N 3°41'04.3"E	*Pulicaria undulata*	Guertoufa (قرطوفة، جثجاث)	Asteraceae	It has been traditionally used to treat diabetes, cardiac disorders, skin diseases, abscesses, inflammations, an insect repellent as well as an herbal tea and tonic. Several investigations have<br>reported the chemical composition, antibacterial and antioxidant activities of the essential oil of *P. undulata* [25-29].
5	Messaad, Djelfa	34°06'07.8"N 3°33'46.4"E	*Arthrophytum scoparium*	Remth (رمث)	Amaranthaceae	It is used as a traditional medicine for the treatment of ocular disorders, against mold, for its hepatoprotective, antioxidant, antitumor and anti-larval properties [30]. It exerts potent anti-amnesic effects [31].
6	Messaad, Djelfa	34°06'02.4"N 3°33'56.1"E	*Astragalus armatus*	Guendal (قندال، قتاد)	Fabaceae	It is used to treat leischamaniose and helminthiasis [22]. It is used against chronic bronchitis, stomach ulcer, cough, hypertension, diabetes, gynecological disorders and poisonous scorpion stings [32].

	Messaad, Djelfa	34°06'12.6"N 3°33'49.8"E	*Retama raetam*	R'tem (رتم)	Fabaceae	Retama species contain alkaloids, flavonoids with antibacterial, antifungal and cytotoxic activities [33]. *Retama raetam* is widely used in dune stabilization and soil fixation. Furthermore, it is capable of producing significant quantities of biomass which are exploited as fodder, an important raw material resource for microfibers production and for the treatment of various human and animal diseases as intestinal worms, scabies, Fever, abortion [22, 34].
8	Ain Naga, Djelfa	34°24'18.1"N 3°28'38.2"E	*Stipa tenacissima*	Halfa (حلفاء)	Poaceae	It is also named Esparto grass and in Algeria it is used as a main source of fiber for paper production [35]. It is used to modulate melancholy, neuralgia and hypercholesterolemia [22].
9	Moudjbara, Djelfa	34°34'19.3"N 3°25'28.2"E	*Artemisia herba-alba*	Shih (شيح)	Asteraceae	It is used in the traditional medicine of the Northern Badia region of Jordan, in the form of a decoction, against fever and menstrual and nervous problems. It is also known as desert wormwood and it has been used in folk medicine by many cultures since ancient times and in Moroccan folk medicine to treat arterial hypertension and/or diabetes [36]. In Algeria, it is used to treat stomach pain and some genital infections [22].
10	Zaafrane, Djelfa	34°52'04.6"N 2°50'56.5"E	*Salsola tetragona*	Djel, Belbel (جل)	Chenopodiaceae	The leaves and sprouts of many *Salsola* species are edible, and sometimes the plants are specially grown and used for salads or to flavor sushi. It is used also as camel forage [37]. Furthermore, it is used for the treatment of indigestion, constipation, belly and stomach pain [38].
11	Zaafrane, Djelfa	34°52'09.7"N 2°50'56.7"E	*Atriplex halimus*	G'taf (قطف)	Chenopodiaceae	It has a high agricultural value: the leaves, available all year round, have an excellent salty taste and can be added to salads or cooked like spinach, while the seeds are used to thicken soups or are mixed with cereals to make bread. It is the common fodder shrub that are used for revegetation of rangelands and to generate plant cover in contaminated soils [39]. The main curative aspect is the treatment of cysts [40].
12	Zaafrane, Djelfa	34°52'19.3"N 2°50'58.3"E	*Peganum harmala*	Harmal, Syrian Rue (حرمل)	Nitrariaceae	It is used for medicinal and psychoactive purposes since ancient times. Its seeds are known to possess hypothermic and hallucinogenic properties, and are used as spices or condiments. Due to its abortive,

						narcotic, aphrodisiac, stimulant, sedative, and emetic properties it is used for the treatment of syphilis, fever, hysteria, malaria, neuralgia, parkinsonism, rheumatism, colic, asthma and ocular disorders [41]. It is used also to alleviate leishmaniasis, rheumatoid arthritis, seizures and depression [22].
13	Zaafrane, Djelfa	34°52'26.9"N 2°50'53.4"E	*Suaeda fruticosa*	Suaeda (سويداء)	Amaranthaceae	It is a source of cardiotonic and anti-infective agents. It could be a valuable source of phenolic and flavonoid compounds with antioxidant, anti-inflammatory and anticancer properties [42].
14	Zaafrane, Djelfa	34°52'01.1"N 2°50'56.6"E	*Thymelaea microphylla*	Methnan (مثنان)	Thymeleaceae	It shows biological activities thanks to the richness of bioactive secondary metabolites such as flavonoids, sterols, terpeness and coumarins [43]. It is used for hair care and the alleviation of hair loss, helminthiasis and depression [22].

Table S2. Physical-chemical properties of the soils sampled from the rhizosphere of 14 different plant species in North-Central Algeria.

**Area**	**Texture**	**pH**	**Organic matter (%)**	**Total $CaCO_3$ (%)**	**Active $CaCO_3$ (%)**	**Total Phosphorus (ppm)**	**Olsen Phosphorus (ppm)**	**Electrical conductivity (Ms/cm) at 25°C**	**Total Nitrogen (%)**	**Plant species**
**Metlili, Ghardaïa**	Sandy-silty	7.2±0.06	0.33±0.01	8.87±0.18	3.31±0.13	102.54±2.88	8.13±0.20	0.525±0.02	0.13±0.01	*Cleome arabica*<br>*Reseda villosa*
**Beni Isguen, Ghardaïa**	Sandy-silty	8.31±0.10	0.89±0.03	7.26±0.20	5.23±0.11	112.39±4.80	19.88±0.26	0.612±0.03	0.15±0.02	*Zilla spinosa*<br>*Pulicaria undulata*
**Messaad, Djelfa**	Silty-sandy	8.23±0.07	0.36±0.01	10.89±0.09	4.53±0.09	138.07±3.20	12.27±0.16	0.416±0.01	0.07±0.01	*Arthrophytum scoparium*<br>*Astragalus armatus*<br>*Retama raetam*
**Ain Naga, Djelfa**	Silty-sandy	7.83±0.05	0.41±0.02	12.19±0.19	6.11±0.05	148.78±3.50	33.22±0.20	0.211±0.01	0.08±0.01	*Stipa tenacissima*
**Moudjbara, Djelfa**	Silty-sandy	7.23±0.05	0.45±0.04	11.5±0.07	5.65±0.04	168.02±4.11	24.56±0.30	0.287±0.02	0.12±0.02	*Artemisia herba-alba*
**Zaafrane, Djelfa**	Sandy-silty	7.9±0.07	0.51±0.03	9.56±0.13	4.12±0.05	152.31±3.89	3.54±0.05	0.823±0.02	0.11±0.01	*Salsola tetragona*<br>*Atriplex halimus*<br>*Peganum harmala*<br>*Suaeda fruticosa*<br>*Thymelaea microphylla*
**Method***	Soil Survey Staff (1999)	Al-Busaidi et al. (2005)	Baize and Jabiol (2011)	Baize and Jabiol (2011)	Baize and Jabiol (2011)	Rowland and Haygarth (1997)	Maghsoodi et al. (2015)	Thomas (1982)	Bremner and Tabatabai (1972)	

Maghsoodi, M., Reyhanitabar, A. and Najafi, N. (2015). Development of an alternative to the Olsen test for determining corn plant-available phosphorus in calcareous soils. *Iran Agricultural Research*, 34(1), 92-104. doi: 10.22099/iar.2015.3096

Rowland, A.P. and Haygarth, P.M. (1997). Determination of total dissolved phosphorus in soil solutions. Journal of Environmental Quality, 26: 410–415. doi: 10.2134/jeq1997.00472425002600020011x

Soil Survey Staff (1999). Soil Taxonomy: A Basic System of Soil Classification for Making and Interpreting Soil Surveys, second ed. Natural Resources Conservation Service, U.S. Department of Agriculture, Washington, DC. Agriculture Handbook 436.

Thomas, G.W. (1982). Exchangeable cations. In: A.L. Page, R.H. Muler, and D.R. Keeney, editors. Methods of soil analysis. Part 2. Agron. Monogr. 9. 2nd ed. ASA and SSSA, Madison, WI. p. 159-164.

Table S3. Linear discriminant analysis Effect Size (LEfSe) at species level according to sampling site. All the possible comparisons are reported in the table. Light gray indicates species less present in the first sampling site than in the second one and red indicates a presence less than 50%. Dark gray indicates species more present in the first sampling site than in the second one and green indicates a presence more than 200%.

Species	Phylum	P-values	LDA score	Beni Isguen/ Metlili %	Beni Isguen/ Ain Naga %	Beni Isguen/ Messad %	Beni Isguen/ Moudjbara %	Beni Isguen/ Zaafrane %	Metlili/ Ain Naga %	Metlili/ Messad %	Metlili/ Moudjbara %	Metlili/ Zaafrane %	Ain Naga/ Messad %	Ain Naga/ Moudjbara %	Ain Naga/ Zaafrane %	Messad/ Moudjbara %	Messad/ Zaafrane %	Moudjbara/ Zaafrane %
unclassified_Acidobacteria	Acidobacteria	0.0002639	4.25	98%	117%	153%	71%	150%	119%	157%	72%	153%	131%	61%	128%	46%	98%	212%
unclassified_Acidobacteria_Gp3	Acidobacteria	0.0000008	4.27	169%	153%	471%	196%	481%	91%	279%	116%	285%	308%	128%	314%	41%	102%	246%
unclassified_Acidobacteria_Gp4	Acidobacteria	0.0000001	4.70	23%	71%	21%	10%	29%	316%	92%	46%	127%	29%	15%	40%	50%	138%	276%
unclassified_Blastocatella	Acidobacteria	0.0035542	4.11	17%	39%	28%	9%	27%	227%	159%	54%	156%	70%	24%	69%	34%	98%	288%
Rubrobacter_radiotolerans	Actinobacteria	0.0040006	3.96	11%	44%	21%	14%	26%	403%	188%	124%	239%	47%	31%	59%	66%	127%	192%
unclassified_Acidimicrobiales	Actinobacteria	0.0032255	4.51	177%	157%	160%	211%	157%	89%	90%	119%	89%	102%	134%	100%	132%	98%	74%
unclassified_Aciditerrimonas	Actinobacteria	0.0000362	4.57	336%	569%	434%	645%	675%	169%	129%	192%	201%	76%	113%	119%	149%	155%	105%
unclassified_Actinobacteria	Actinobacteria	0.0000002	5.74	194%	491%	160%	304%	358%	253%	82%	157%	185%	33%	62%	73%	190%	224%	118%
unclassified_Actinomycetales	Actinobacteria	0.0000744	5.08	154%	116%	153%	219%	166%	75%	99%	142%	108%	132%	189%	143%	143%	109%	76%
unclassified_Blastococcus	Actinobacteria	0.0000242	4.08	77%	25%	206%	39%	112%	33%	267%	51%	144%	820%	156%	444%	19%	54%	284%
unclassified_Euzebya	Actinobacteria	0.0000020	4.06	694%	746%	1657%	1435%	182%	107%	239%	207%	26%	222%	192%	24%	87%	11%	13%
unclassified_Gaiella	Actinobacteria	0.0000029	5.31	484%	2824%	346%	420%	2188%	583%	71%	87%	452%	12%	15%	77%	121%	633%	521%
unclassified_Geodermatophilaceae	Actinobacteria	0.0032241	3.84	67%	51%	94%	81%	43%	77%	142%	121%	65%	185%	158%	85%	86%	46%	54%
unclassified_Geodermatophilus	Actinobacteria	0.0000882	3.94	31%	23%	128%	49%	47%	75%	415%	158%	152%	553%	211%	203%	38%	37%	96%
unclassified_Ilumatobacter	Actinobacteria	0.0014008	4.19	65%	27%	53%	15%	36%	41%	80%	23%	55%	196%	55%	134%	28%	68%	243%
unclassified_Microbacteriaceae	Actinobacteria	0.0000283	4.20	146%	23%	100%	139%	48%	16%	69%	96%	33%	440%	615%	211%	140%	48%	34%
unclassified_Micrococcaceae	Actinobacteria	0.0000402	4.43	172%	312%	307%	147%	93%	181%	178%	85%	54%	99%	47%	30%	48%	30%	63%
unclassified_Micromonosporaceae	Actinobacteria	0.0000011	4.64	56%	38%	107%	86%	121%	67%	189%	152%	214%	281%	226%	317%	80%	113%	140%
unclassified_Nocardioidaceae	Actinobacteria	0.0000001	4.49	365%	129%	237%	354%	158%	35%	65%	97%	43%	184%	275%	123%	150%	67%	45%
unclassified_Nocardioides	Actinobacteria	0.0007040	4.12	250%	85%	247%	266%	195%	34%	99%	106%	78%	290%	312%	228%	108%	79%	73%
unclassified_Propionibacteriaceae	Actinobacteria	0.0000991	4.20	9%	37%	25%	22%	81%	420%	282%	254%	933%	67%	60%	222%	90%	331%	368%
unclassified_Pseudonocardiaceae	Actinobacteria	0.0024329	4.08	125%	138%	108%	378%	191%	111%	87%	304%	153%	78%	274%	138%	350%	177%	51%
unclassified_Rubrobacter	Actinobacteria	0.0000019	5.25	17%	22%	16%	9%	19%	127%	91%	50%	112%	72%	39%	89%	55%	123%	224%
unclassified_Solirubrobacter	Actinobacteria	0.0000282	4.10	182%	215%	312%	281%	518%	118%	172%	155%	286%	146%	131%	242%	90%	166%	184%
unclassified_Solirubrobacterales	Actinobacteria	0.0000732	4.61	171%	219%	154%	183%	244%	128%	91%	107%	143%	71%	83%	112%	118%	158%	134%
unclassified_Streptomycetaceae	Actinobacteria	0.0054961	3.97	191%	184%	65%	293%	200%	96%	34%	153%	105%	36%	160%	109%	448%	305%	68%
unclassified_Thermoleophilum	Actinobacteria	0.0000001	4.01	279%	699%	187%	456%	991%	251%	67%	164%	356%	27%	65%	142%	244%	530%	218%
unclassified_Archaea	Archaea	0.0001193	3.77	105%	115%	168%	97%	301%	109%	160%	92%	286%	147%	85%	262%	58%	179%	310%
unclassified_Armatimonadetes_gp4	Armatimonadetes	0.0000001	3.97	131%	428%	251%	284%	480%	328%	192%	218%	367%	59%	66%	112%	113%	191%	169%
unclassified_Armatimonas1Armatimonadetes_gp1	Armatimonadetes	0.0000833	3.92	34%	54%	28%	31%	24%	162%	84%	91%	71%	52%	56%	44%	109%	85%	78%
Adhaeribacter_aquaticus_(T)	Bacteroidetes	0.0000136	3.95	704%	162%	1430%	704%	274%	23%	203%	100%	39%	884%	436%	170%	49%	19%	39%
unclassified_Adhaeribacter	Bacteroidetes	0.0000003	4.14	787%	203%	1593%	592%	341%	26%	203%	75%	43%	783%	291%	168%	37%	21%	58%
unclassified_Bacteroidetes	Bacteroidetes	0.0000001	4.98	56%	15%	35%	23%	13%	27%	62%	42%	24%	232%	158%	90%	68%	39%	57%
unclassified_Chitinophagaceae	Bacteroidetes	0.0125190	5.02	141%	86%	123%	69%	97%	61%	87%	49%	69%	143%	80%	113%	56%	79%	140%
unclassified_Cytophagaceae	Bacteroidetes	0.0000044	3.95	634%	124%	439%	260%	130%	20%	69%	41%	20%	354%	209%	105%	59%	30%	50%
unclassified_Cytophagales	Bacteroidetes	0.0000005	3.87	120%	28%	273%	68%	30%	24%	226%	57%	25%	958%	240%	105%	25%	11%	44%
unclassified_Flavobacteriaceae	Bacteroidetes	0.0000000	4.49	186%	3%	34%	26%	2%	1%	18%	14%	1%	1258%	973%	62%	77%	5%	6%
unclassified_Ohtaekwangia	Bacteroidetes	0.0000037	5.34	47%	11%	71%	40%	31%	23%	151%	85%	65%	655%	370%	284%	56%	43%	77%
unclassified_Sphingobacteriaceae	Bacteroidetes	0.0000001	3.99	95%	72%	433%	151%	59%	76%	456%	159%	62%	599%	209%	82%	35%	14%	39%
unclassified_Saccharibacteria_genera_incertae_sedis	Candidatus Saccharibacteria	0.0000000	4.97	34%	10%	55%	56%	22%	30%	161%	166%	64%	546%	564%	215%	103%	39%	38%
unclassified_Anaerolineaceae	Chloroflexi	0.0000000	5.00	189%	475%	957%	1611%	797%	252%	507%	854%	423%	202%	339%	168%	168%	83%	50%
unclassified_Chloroflexi	Chloroflexi	0.0000001	4.83	77%	74%	256%	107%	199%	97%	334%	140%	260%	345%	145%	268%	42%	78%	185%
unclassified_Litorilinea	Chloroflexi	0.0000811	3.74	97%	60%	687%	201%	118%	61%	706%	207%	121%	1152%	337%	197%	29%	17%	58%
unclassified_Sphaerobacter	Chloroflexi	0.0006056	3.79	82%	78%	223%	245%	151%	96%	273%	301%	185%	286%	315%	194%	110%	68%	62%
unclassified_Thermomicrobia	Chloroflexi	0.0006216	4.02	64%	36%	212%	41%	65%	56%	330%	64%	102%	593%	114%	183%	19%	31%	160%
unclassified_Bacillales	Firmicutes	0.0000011	4.21	178%	243%	572%	355%	209%	137%	322%	200%	118%	235%	146%	86%	62%	37%	59%
unclassified_Gemmatimonas	Gemmatimonadetes	0.0002522	5.13	59%	51%	71%	24%	39%	87%	120%	42%	67%	139%	48%	77%	35%	56%	160%
unclassified_Gemmata	Planctomycetes	0.0000377	3.83	65%	118%	136%	45%	208%	181%	208%	70%	319%	115%	38%	176%	33%	153%	458%
unclassified_Pirellula	Planctomycetes	0.0000000	4.47	206%	150%	464%	485%	557%	73%	225%	235%	270%	309%	323%	371%	104%	120%	115%
unclassified_Planctomyces	Planctomycetes	0.0000026	4.20	16%	9%	42%	37%	15%	53%	261%	231%	95%	492%	434%	179%	88%	36%	41%
unclassified_Planctomycetaceae	Planctomycetes	0.0000036	5.14	68%	56%	120%	133%	109%	82%	177%	197%	161%	215%	239%	196%	111%	91%	82%
unclassified_Planctomycetes	Planctomycetes	0.0000197	3.65	70%	78%	306%	189%	158%	113%	440%	271%	228%	391%	241%	202%	62%	52%	84%
gamma_proteobacterium_SA29_B	Proteobacteria	0.0000539	4.08	39%	9%	40%	157%	27%	24%	104%	406%	68%	429%	1678%	283%	392%	66%	17%
unclassified_Acetobacteraceae	Proteobacteria	0.0137480	3.79	286%	138%	198%	187%	225%	48%	69%	65%	79%	143%	135%	163%	95%	114%	120%
unclassified_Alphaproteobacteria	Proteobacteria	0.0000104	4.84	48%	37%	46%	75%	38%	76%	97%	157%	80%	127%	205%	105%	162%	83%	51%
unclassified_Betaproteobacteria	Proteobacteria	0.0009143	4.66	121%	109%	162%	83%	149%	90%	133%	69%	123%	148%	76%	136%	52%	92%	179%
unclassified_Bradyrhizobiaceae	Proteobacteria	0.0000009	3.87	16%	15%	16%	19%	33%	97%	100%	120%	206%	103%	123%	213%	120%	207%	172%
unclassified_Burkholderiales	Proteobacteria	0.0000374	4.43	75%	201%	360%	188%	207%	269%	482%	252%	278%	179%	94%	103%	52%	58%	110%
unclassified_Caulobacteraceae	Proteobacteria	0.0000393	3.91	71%	15%	27%	33%	18%	21%	38%	46%	26%	177%	216%	120%	122%	68%	55%
unclassified_Comamonadaceae	Proteobacteria	0.0000071	4.22	430%	221%	406%	132%	133%	51%	94%	31%	31%	184%	60%	60%	33%	33%	101%
unclassified_Cystobacteraceae	Proteobacteria	0.0000874	4.11	32%	92%	124%	85%	143%	287%	388%	265%	446%	135%	93%	156%	68%	115%	168%
unclassified_Deltaproteobacteria	Proteobacteria	0.0001865	4.11	150%	155%	284%	203%	208%	103%	189%	135%	138%	184%	131%	134%	71%	73%	103%
unclassified_Erythrobacteraceae	Proteobacteria	0.0000000	4.34	73%	35%	37%	59%	6%	48%	51%	82%	8%	107%	171%	16%	160%	15%	9%
unclassified_Gammaproteobacteria	Proteobacteria	0.0000004	4.79	51%	20%	57%	102%	40%	38%	110%	199%	78%	287%	519%	204%	181%	71%	39%
unclassified_Hyphomicrobiaceae	Proteobacteria	0.0002941	3.64	93%	65%	68%	224%	104%	70%	73%	241%	112%	104%	343%	159%	329%	153%	47%
unclassified_Microvirga	Proteobacteria	0.0000735	4.45	35%	44%	259%	132%	115%	126%	739%	376%	326%	587%	299%	260%	51%	44%	87%
unclassified_Myxococcales	Proteobacteria	0.0005790	4.63	47%	39%	90%	55%	52%	83%	189%	116%	110%	228%	140%	132%	61%	58%	95%
unclassified_Nitrospira	Proteobacteria	0.0000002	3.79	128%	149%	187%	142%	475%	116%	146%	110%	370%	125%	95%	318%	76%	254%	335%
unclassified_Oxalobacteraceae	Proteobacteria	0.0004758	4.56	695%	274%	580%	434%	281%	39%	83%	62%	40%	212%	159%	103%	75%	48%	65%

Species	Phylum	P-values	LDA score	Beni Isguen/ Metlili %	Beni Isguen/ Ain Naga %	Beni Isguen/ Messaad %	Beni Isguen/ Moudjbara %	Beni Isguen/ Zaafrane %	Metlili/ Ain Naga %	Metlili/ Messaad %	Metlili/ Moudjbara %	Metlili/ Zaafrane %	Ain Naga/ Messaad %	Ain Naga/ Moudjbara %	Ain Naga/ Zaafrane %	Messad/ Moudjbara %	Messad/ Zaafrane %	Moudjbara/ Zaafrane %
unclassified_Phaselicystis	Proteobacteria	0.0000023	3.85	12%	4%	16%	13%	8%	33%	135%	110%	71%	407%	332%	215%	82%	53%	65%
unclassified_Phenylobacterium	Proteobacteria	0.0026468	4.14	114%	23%	139%	127%	127%	20%	123%	112%	112%	619%	564%	563%	91%	91%	100%
unclassified_Phyllobacteriaceae	Proteobacteria	0.0000378	3.95	52%	9%	34%	129%	29%	17%	66%	249%	57%	382%	1449%	329%	379%	86%	23%
unclassified_Proteobacteria	Proteobacteria	0.0006293	4.42	69%	66%	106%	129%	81%	95%	153%	186%	117%	160%	195%	123%	122%	77%	63%
unclassified_Rhizobiales	Proteobacteria	0.0000014	5.05	40%	35%	82%	71%	68%	87%	203%	176%	168%	233%	202%	193%	87%	83%	95%
unclassified_Rhodobacteraceae	Proteobacteria	0.0000389	3.78	264%	222%	485%	543%	122%	84%	184%	206%	46%	218%	245%	55%	112%	25%	23%
unclassified_Rhodospirillaceae	Proteobacteria	0.0000000	4.22	35%	50%	73%	181%	33%	144%	212%	523%	94%	147%	362%	65%	246%	44%	18%
unclassified_Rhodospirillales	Proteobacteria	0.0000069	3.90	69%	73%	142%	284%	120%	106%	206%	410%	173%	194%	386%	163%	199%	84%	42%
unclassified_Rubellimicrobium	Proteobacteria	0.0000026	4.61	443%	302%	1579%	901%	1179%	68%	356%	203%	266%	522%	298%	390%	57%	75%	131%
unclassified_Skermanella	Proteobacteria	0.0030325	4.13	116%	48%	388%	125%	50%	42%	335%	108%	43%	802%	259%	103%	32%	13%	40%
unclassified_Sphingomonadaceae	Proteobacteria	0.0001146	4.70	76%	58%	70%	93%	43%	77%	92%	124%	56%	119%	160%	73%	134%	61%	46%
unclassified_Sphingomonadales	Proteobacteria	0.0000053	4.13	104%	44%	61%	108%	31%	42%	59%	104%	30%	139%	247%	72%	178%	52%	29%
unclassified_Sphingomonas	Proteobacteria	0.0014753	4.07	144%	89%	195%	128%	192%	62%	135%	89%	133%	220%	144%	217%	66%	99%	150%
unclassified_Sphingosinicella	Proteobacteria	0.0027862	4.09	52%	50%	207%	51%	99%	96%	396%	96%	190%	414%	101%	198%	24%	48%	197%
unclassified_Steroidobacter	Proteobacteria	0.0000032	4.42	13%	6%	22%	26%	19%	43%	166%	198%	147%	388%	464%	346%	119%	89%	75%
unclassified_Xanthomonadaceae	Proteobacteria	0.0000000	4.64	33%	5%	30%	90%	10%	14%	91%	270%	29%	632%	1869%	201%	296%	32%	11%
bacterium_Ellin6510	unclassified_Bacteria	0.0000312	4.71	957%	6732%	262%	944%	816%	703%	27%	99%	85%	4%	14%	12%	361%	312%	86%
unclassified_Bacteria	unclassified_Bacteria	0.0001553	5.96	82%	103%	67%	82%	75%	125%	81%	99%	91%	65%	79%	73%	122%	112%	91%
unclassified_Gp10	unclassified_Bacteria	0.0000154	4.15	10%	23%	72%	72%	41%	237%	758%	753%	433%	319%	317%	182%	99%	57%	57%
unclassified_Gp3	unclassified_Bacteria	0.0001731	4.06	200%	200%	321%	270%	254%	100%	160%	135%	127%	161%	135%	127%	84%	79%	94%
unclassified_Gp4	unclassified_Bacteria	0.0001146	4.71	65%	281%	59%	42%	73%	433%	91%	64%	113%	21%	15%	26%	71%	124%	175%
unclassified_Gp7	unclassified_Bacteria	0.0007332	4.28	108%	187%	261%	103%	168%	174%	242%	95%	156%	139%	55%	90%	39%	64%	163%
unclassified_Parcubacteria_genera_incertae_sedis	unclassified_Bacteria	0.0012838	3.66	30%	20%	20%	18%	11%	67%	69%	61%	38%	102%	90%	56%	88%	55%	62%
unclassified_Subdivision3_genera_incertae_sedis	unclassified_Bacteria	0.0005943	4.67	119%	136%	250%	148%	189%	114%	209%	123%	158%	184%	109%	139%	59%	76%	128%
unclassified_WPS_1_genera_incertae_sedis	unclassified_Bacteria	0.0000009	5.32	35%	42%	32%	17%	26%	118%	91%	48%	73%	77%	41%	62%	53%	80%	151%
unclassified_WPS_2_genera_incertae_sedis	unclassified_Bacteria	0.0003330	4.27	131%	360%	220%	189%	259%	275%	169%	145%	198%	61%	53%	72%	86%	117%	137%
unclassified_Opitutus	Verrucomicrobia	0.0023655	4.32	111%	53%	191%	65%	67%	47%	171%	59%	60%	363%	124%	128%	34%	35%	103%
unclassified_Spartobacteria_genera_incertae_sedis	Verrucomicrobia	0.0000780	4.47	49%	45%	43%	14%	38%	91%	88%	28%	78%	97%	31%	86%	32%	88%	275%
unclassified_Verrucomicrobia	Verrucomicrobia	0.0007743	4.37	70%	47%	49%	44%	38%	67%	71%	63%	55%	105%	93%	82%	89%	78%	87%

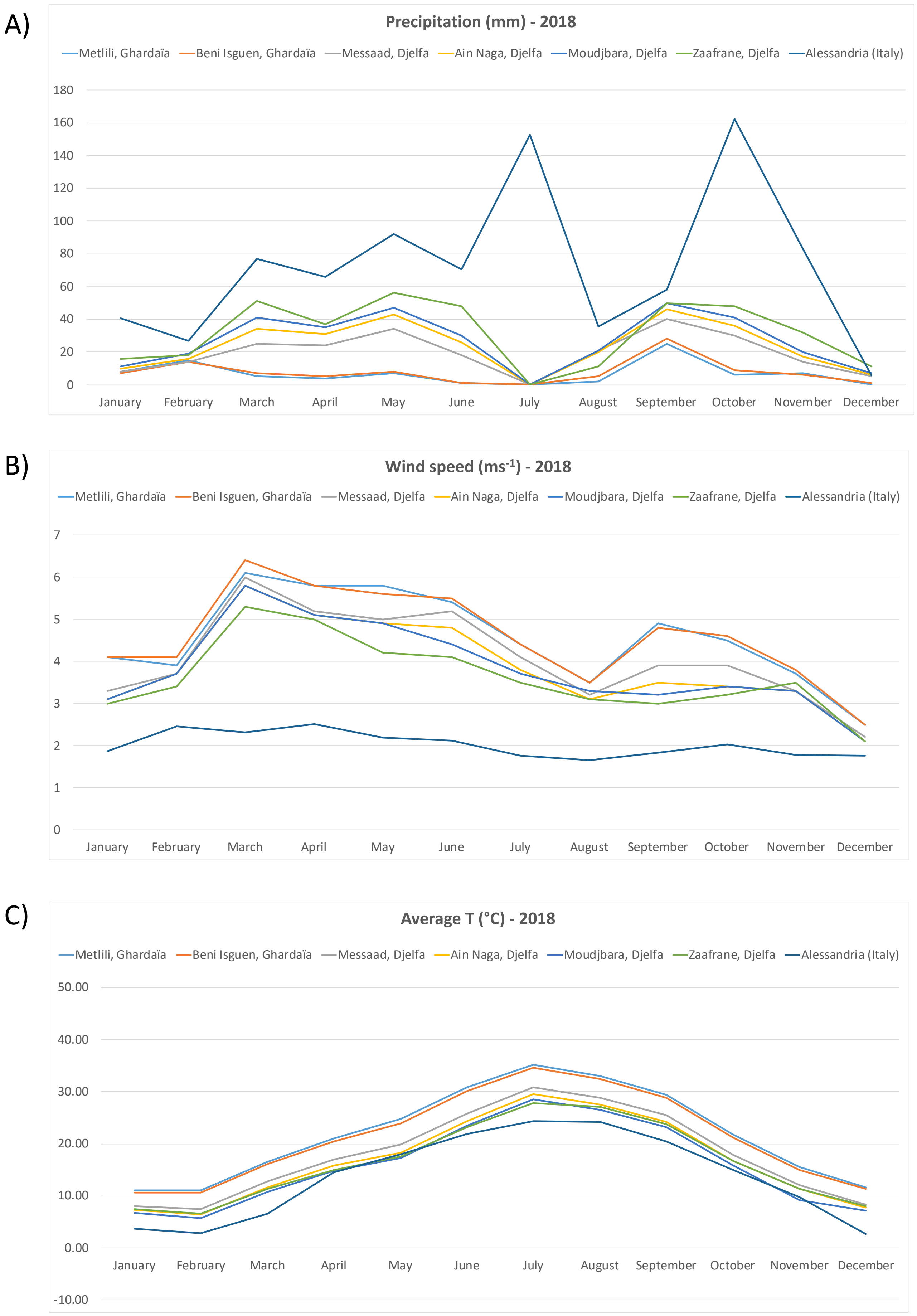
A)
Precipitation (mm) - 2018
Metlili, Ghardaïa
Beni Isguen, Ghardaïa
Messaad, Djelfa
Ain Naga, Djelfa
Moudjbara, Djelfa
Zaafrane, Djelfa
Alessandria (Italy)
180
160
140
120
100
80
60
40
20
0
January February March April May June July August September October November December
B)
Wind speed (ms-1) - 2018
Metlili, Ghardaïa
Beni Isguen, Ghardaïa
Messaad, Djelfa
Ain Naga, Djelfa
Moudjbara, Djelfa
Zaafrane, Djelfa
Alessandria (Italy)
7
6
5
4
3
2
1
0
January February March April May June July August September October November December
C)
Average T (°C) - 2018
Metlili, Ghardaïa
Beni Isguen, Ghardaïa
Messaad, Djelfa
Ain Naga, Djelfa
Moudjbara, Djelfa
Zaafrane, Djelfa
Alessandria (Italy)
50.00
40.00
30.00
20.00
10.00
0.00
-10.00
January February March April May June July August September October November December

Figure S1.

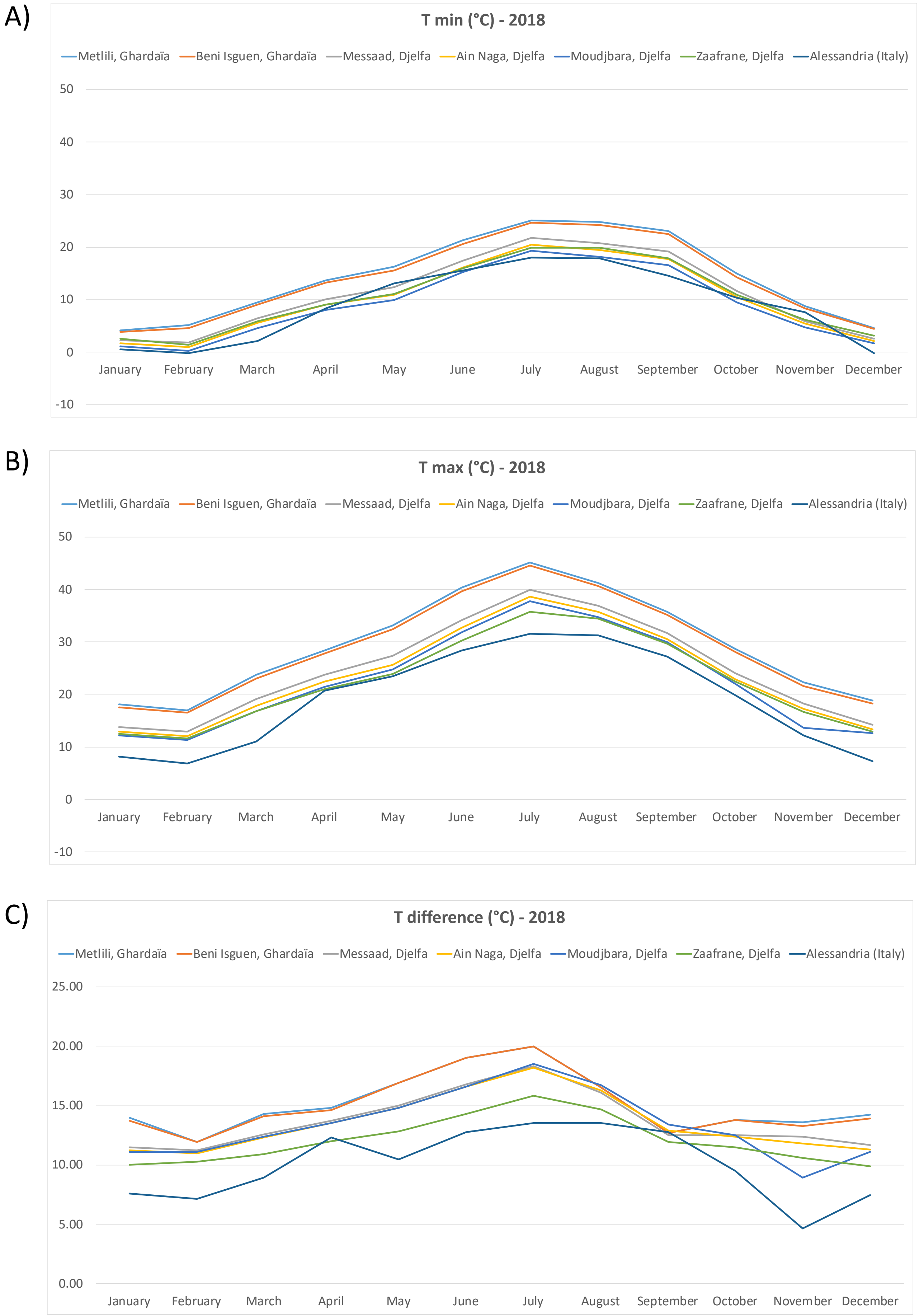
A)
T min (°C) - 2018
Metlili, Ghardaïa
Beni Isguen, Ghardaïa
Messaad, Djelfa
Ain Naga, Djelfa
Moudjbara, Djelfa
Zaafrane, Djelfa
Alessandria (Italy)
50
40
30
20
10
0
-10
January
February
March
April
May
June
July
August
September
October
November
December
B)
T max (°C) - 2018
Metlili, Ghardaïa
Beni Isguen, Ghardaïa
Messaad, Djelfa
Ain Naga, Djelfa
Moudjbara, Djelfa
Zaafrane, Djelfa
Alessandria (Italy)
50
40
30
20
10
0
-10
January
February
March
April
May
June
July
August
September
October
November
December
C)
T difference (°C) - 2018
Metlili, Ghardaïa
Beni Isguen, Ghardaïa
Messaad, Djelfa
Ain Naga, Djelfa
Moudjbara, Djelfa
Zaafrane, Djelfa
Alessandria (Italy)
25.00
20.00
15.00
10.00
5.00
0.00
January
February
March
April
May
June
July
August
September
October
November
December

Figure S2.

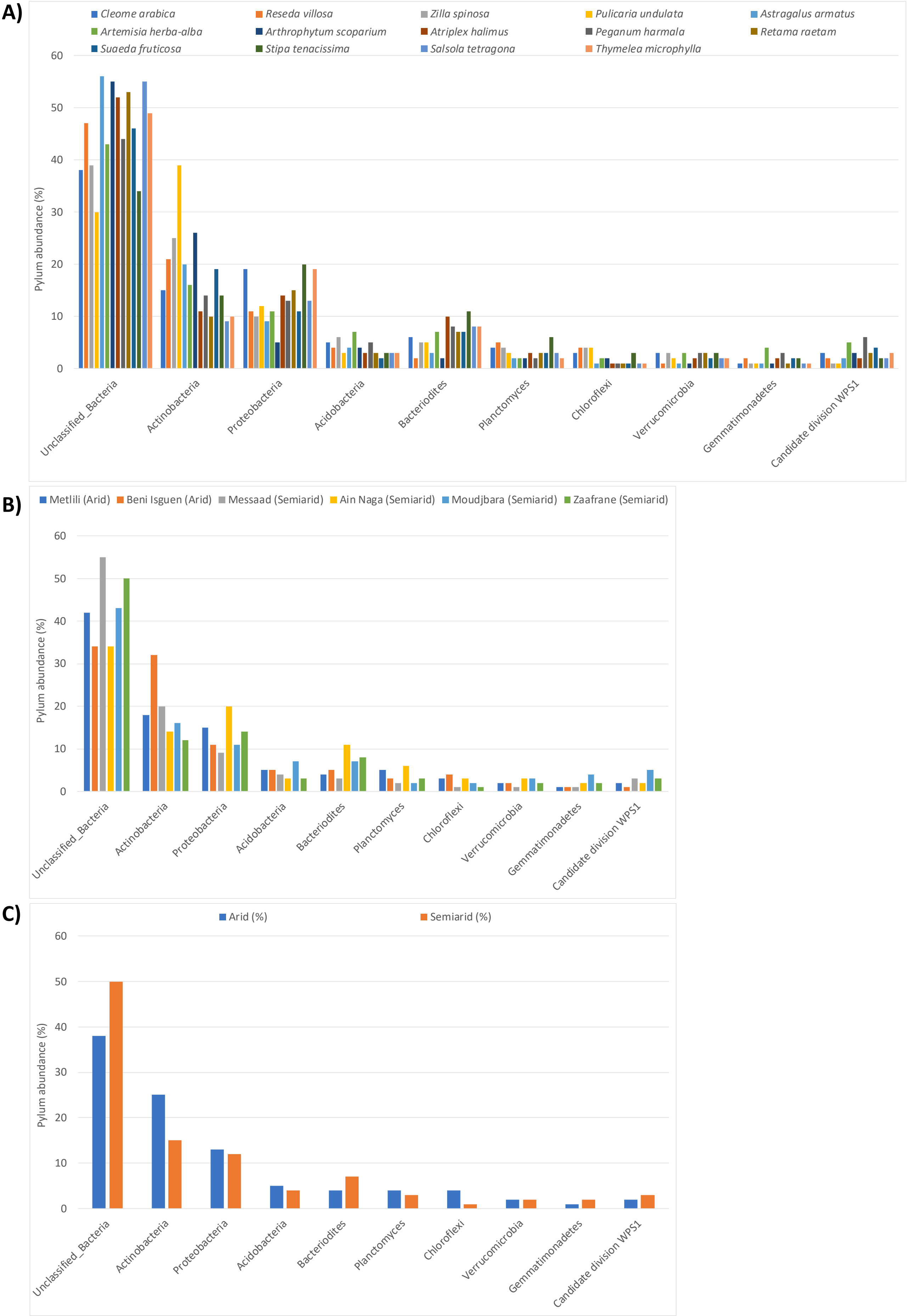
A)
Cleome arabica
Reseda villosa
Zilla spinosa
Pulicaria undulata
Astragalus armatus
Artemisia herba-alba
Arthrophytum scoparium
Atriplex halimus
Peganum harmala
Retama raetam
Suaeda fruticosa
Stipa tenacissima
Salsola tetragona
Thymelea microphylla
Pylum abundance (%)
Unclassified_Bacteria
Actinobacteria
Proteobacteria
Acidobacteria
Bacteriodites
Planctomyces
Chloroflexi
Verrucomicrobia
Gemmatimonadetes
Candidate division WPS1
B)
Metlili (Arid)
Beni Isguen (Arid)
Messaad (Semiarid)
Ain Naga (Semiarid)
Moudjbara (Semiarid)
Zaafrane (Semiarid)
C)
Arid (%)
Semiarid (%)

Figure S3.

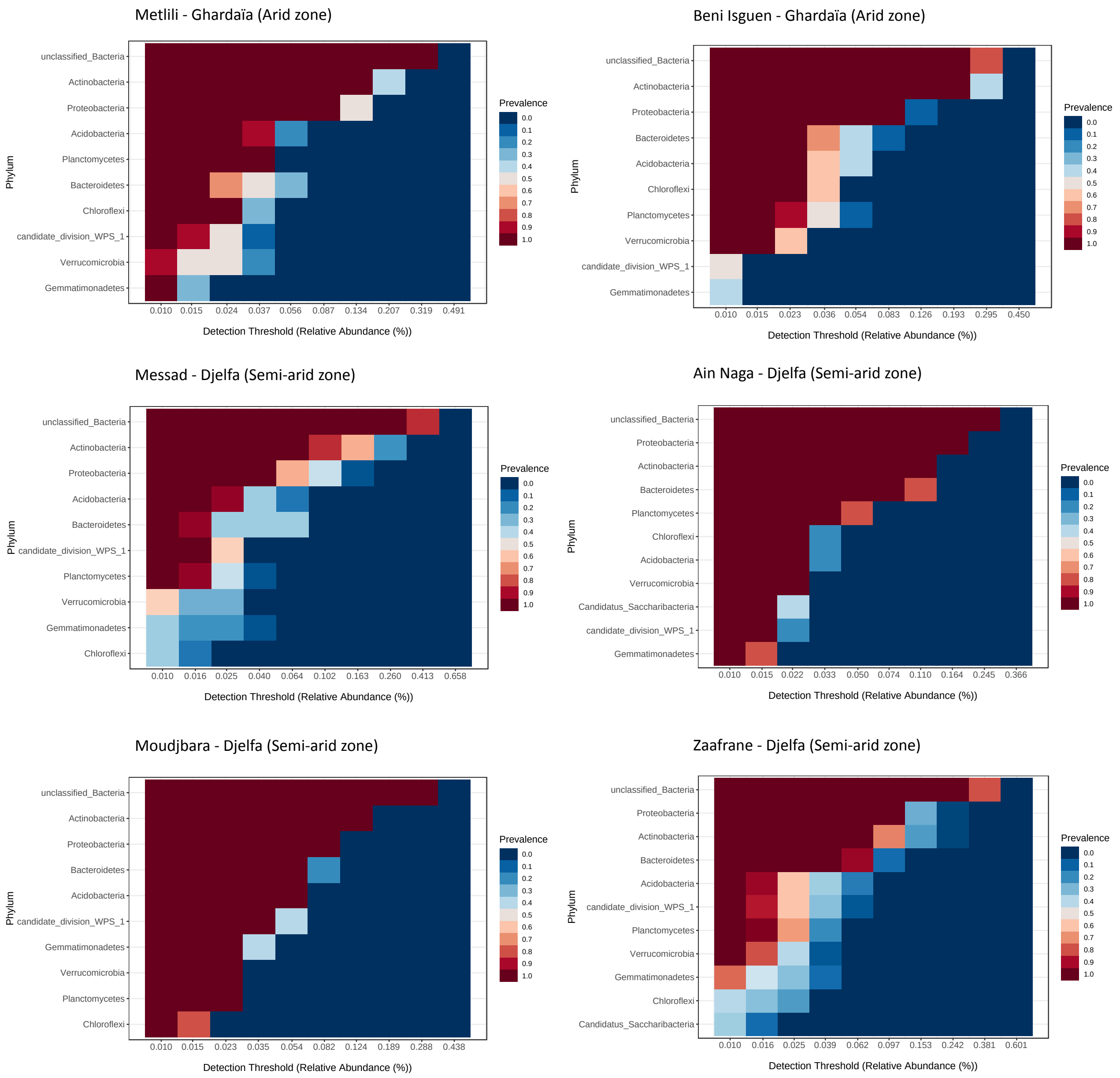
Metlili - Ghardaïa (Arid zone)
unclassified_Bacteria
Actinobacteria
Proteobacteria
Acidobacteria
Planctomycetes
Bacteroidetes
Chloroflexi
candidate_division_WPS_1
Verrucomicrobia
Gemmatimonadetes
Phylum
0.010 0.015 0.024 0.037 0.056 0.087 0.134 0.207 0.319 0.491
Detection Threshold (Relative Abundance (%))
Prevalence
0.0 0.1 0.2 0.3 0.4 0.5 0.6 0.7 0.8 0.9 1.0
Beni Isguen - Ghardaïa (Arid zone)
unclassified_Bacteria
Actinobacteria
Proteobacteria
Bacteroidetes
Acidobacteria
Chloroflexi
Planctomycetes
Verrucomicrobia
candidate_division_WPS_1
Gemmatimonadetes
Phylum
0.010 0.015 0.023 0.036 0.054 0.083 0.126 0.193 0.295 0.450
Detection Threshold (Relative Abundance (%))
Prevalence
0.0 0.1 0.2 0.3 0.4 0.5 0.6 0.7 0.8 0.9 1.0
Messad - Djelfa (Semi-arid zone)
unclassified_Bacteria
Actinobacteria
Proteobacteria
Acidobacteria
Bacteroidetes
candidate_division_WPS_1
Planctomycetes
Verrucomicrobia
Gemmatimonadetes
Chloroflexi
Phylum
0.010 0.016 0.025 0.040 0.064 0.102 0.163 0.260 0.413 0.658
Detection Threshold (Relative Abundance (%))
Prevalence
0.0 0.1 0.2 0.3 0.4 0.5 0.6 0.7 0.8 0.9 1.0
Ain Naga - Djelfa (Semi-arid zone)
unclassified_Bacteria
Proteobacteria
Actinobacteria
Bacteroidetes
Planctomycetes
Chloroflexi
Acidobacteria
Verrucomicrobia
Candidatus_Saccharibacteria
candidate_division_WPS_1
Gemmatimonadetes
Phylum
0.010 0.015 0.022 0.033 0.050 0.074 0.110 0.164 0.245 0.366
Detection Threshold (Relative Abundance (%))
Prevalence
0.0 0.1 0.2 0.3 0.4 0.5 0.6 0.7 0.8 0.9 1.0
Moudjbara - Djelfa (Semi-arid zone)
unclassified_Bacteria
Actinobacteria
Proteobacteria
Bacteroidetes
Acidobacteria
candidate_division_WPS_1
Gemmatimonadetes
Verrucomicrobia
Planctomycetes
Chloroflexi
Phylum
0.010 0.015 0.023 0.035 0.054 0.082 0.124 0.189 0.288 0.438
Detection Threshold (Relative Abundance (%))
Prevalence
0.0 0.1 0.2 0.3 0.4 0.5 0.6 0.7 0.8 0.9 1.0
Zaafrane - Djelfa (Semi-arid zone)
unclassified_Bacteria
Proteobacteria
Actinobacteria
Bacteroidetes
Acidobacteria
candidate_division_WPS_1
Planctomycetes
Verrucomicrobia
Gemmatimonadetes
Chloroflexi
Candidatus_Saccharibacteria
Phylum
0.010 0.016 0.025 0.039 0.062 0.097 0.153 0.242 0.381 0.601
Detection Threshold (Relative Abundance (%))
Prevalence
0.0 0.1 0.2 0.3 0.4 0.5 0.6 0.7 0.8 0.9 1.0

Figure S4.

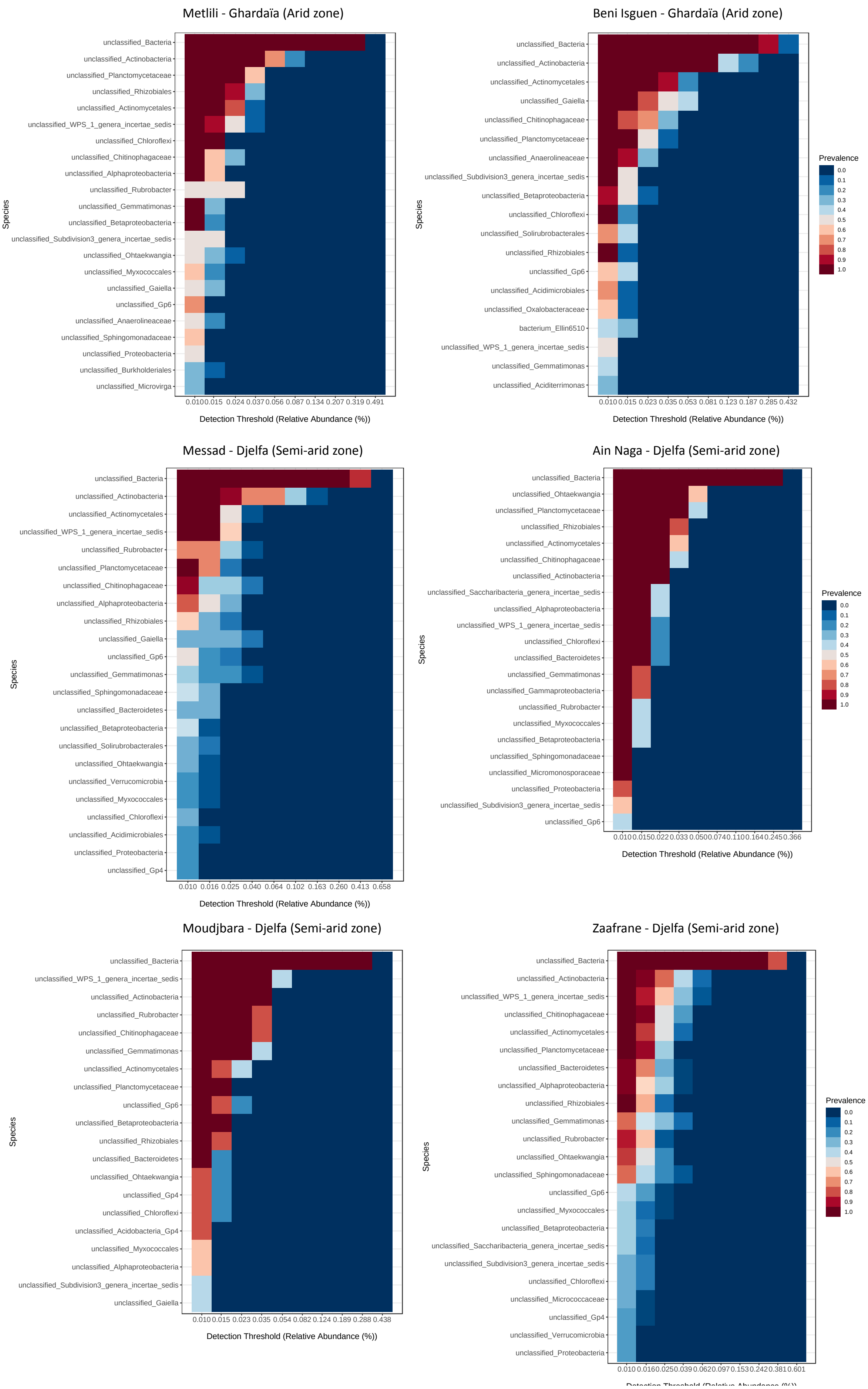

Metlili - Ghardaïa (Arid zone)
Species
unclassified_Bacteria
unclassified_Actinobacteria
unclassified_Planctomycetaceae
unclassified_Rhizobiales
unclassified_Actinomycetales
unclassified_WPS_1_genera_incertae_sedis
unclassified_Chloroflexi
unclassified_Chitinophagaceae
unclassified_Alphaproteobacteria
unclassified_Rubrobacter
unclassified_Gemmatimonas
unclassified_Betaproteobacteria
unclassified_Subdivision3_genera_incertae_sedis
unclassified_Ohtaekwangia
unclassified_Myxococcales
unclassified_Gaiella
unclassified_Gp6
unclassified_Anaerolineaceae
unclassified_Sphingomonadaceae
unclassified_Proteobacteria
unclassified_Burkholderiales
unclassified_Microvirga
0.010 0.015 0.024 0.037 0.056 0.087 0.134 0.207 0.319 0.491
Detection Threshold (Relative Abundance (%))
Beni Isguen - Ghardaïa (Arid zone)
Species
unclassified_Bacteria
unclassified_Actinobacteria
unclassified_Actinomycetales
unclassified_Gaiella
unclassified_Chitinophagaceae
unclassified_Planctomycetaceae
unclassified_Anaerolineaceae
unclassified_Subdivision3_genera_incertae_sedis
unclassified_Betaproteobacteria
unclassified_Chloroflexi
unclassified_Solirubrobacterales
unclassified_Rhizobiales
unclassified_Gp6
unclassified_Acidimicrobiales
unclassified_Oxalobacteraceae
bacterium_Ellin6510
unclassified_WPS_1_genera_incertae_sedis
unclassified_Gemmatimonas
unclassified_Aciditerrimonas
0.010 0.015 0.023 0.035 0.053 0.081 0.123 0.187 0.285 0.432
Detection Threshold (Relative Abundance (%))
Prevalence
0.0
0.1
0.2
0.3
0.4
0.5
0.6
0.7
0.8
0.9
1.0
Messad - Djelfa (Semi-arid zone)
Species
unclassified_Bacteria
unclassified_Actinobacteria
unclassified_Actinomycetales
unclassified_WPS_1_genera_incertae_sedis
unclassified_Rubrobacter
unclassified_Planctomycetaceae
unclassified_Chitinophagaceae
unclassified_Alphaproteobacteria
unclassified_Rhizobiales
unclassified_Gaiella
unclassified_Gp6
unclassified_Gemmatimonas
unclassified_Sphingomonadaceae
unclassified_Bacteroidetes
unclassified_Betaproteobacteria
unclassified_Solirubrobacterales
unclassified_Ohtaekwangia
unclassified_Verrucomicrobia
unclassified_Myxococcales
unclassified_Chloroflexi
unclassified_Acidimicrobiales
unclassified_Proteobacteria
unclassified_Gp4
0.010 0.016 0.025 0.040 0.064 0.102 0.163 0.260 0.413 0.658
Detection Threshold (Relative Abundance (%))
Ain Naga - Djelfa (Semi-arid zone)
Species
unclassified_Bacteria
unclassified_Ohtaekwangia
unclassified_Planctomycetaceae
unclassified_Rhizobiales
unclassified_Actinomycetales
unclassified_Chitinophagaceae
unclassified_Actinobacteria
unclassified_Saccharibacteria_genera_incertae_sedis
unclassified_Alphaproteobacteria
unclassified_WPS_1_genera_incertae_sedis
unclassified_Chloroflexi
unclassified_Bacteroidetes
unclassified_Gemmatimonas
unclassified_Gammaproteobacteria
unclassified_Rubrobacter
unclassified_Myxococcales
unclassified_Betaproteobacteria
unclassified_Sphingomonadaceae
unclassified_Micromonosporaceae
unclassified_Proteobacteria
unclassified_Subdivision3_genera_incertae_sedis
unclassified_Gp6
0.010 0.015 0.022 0.033 0.050 0.074 0.110 0.164 0.245 0.366
Detection Threshold (Relative Abundance (%))
Prevalence
0.0
0.1
0.2
0.3
0.4
0.5
0.6
0.7
0.8
0.9
1.0
Moudjbara - Djelfa (Semi-arid zone)
Species
unclassified_Bacteria
unclassified_WPS_1_genera_incertae_sedis
unclassified_Actinobacteria
unclassified_Rubrobacter
unclassified_Chitinophagaceae
unclassified_Gemmatimonas
unclassified_Actinomycetales
unclassified_Planctomycetaceae
unclassified_Gp6
unclassified_Betaproteobacteria
unclassified_Rhizobiales
unclassified_Bacteroidetes
unclassified_Ohtaekwangia
unclassified_Gp4
unclassified_Chloroflexi
unclassified_Acidobacteria_Gp4
unclassified_Myxococcales
unclassified_Alphaproteobacteria
unclassified_Subdivision3_genera_incertae_sedis
unclassified_Gaiella
0.010 0.015 0.023 0.035 0.054 0.082 0.124 0.189 0.288 0.438
Detection Threshold (Relative Abundance (%))
Zaafrane - Djelfa (Semi-arid zone)
Species
unclassified_Bacteria
unclassified_Actinobacteria
unclassified_WPS_1_genera_incertae_sedis
unclassified_Chitinophagaceae
unclassified_Actinomycetales
unclassified_Planctomycetaceae
unclassified_Bacteroidetes
unclassified_Alphaproteobacteria
unclassified_Rhizobiales
unclassified_Gemmatimonas
unclassified_Rubrobacter
unclassified_Ohtaekwangia
unclassified_Sphingomonadaceae
unclassified_Gp6
unclassified_Myxococcales
unclassified_Betaproteobacteria
unclassified_Saccharibacteria_genera_incertae_sedis
unclassified_Subdivision3_genera_incertae_sedis
unclassified_Chloroflexi
unclassified_Micrococcaceae
unclassified_Gp4
unclassified_Verrucomicrobia
unclassified_Proteobacteria
0.010 0.016 0.025 0.039 0.062 0.097 0.153 0.242 0.381 0.601
Prevalence
0.0
0.1
0.2
0.3
0.4
0.5
0.6
0.7
0.8
0.9
1.0

Figure S5.

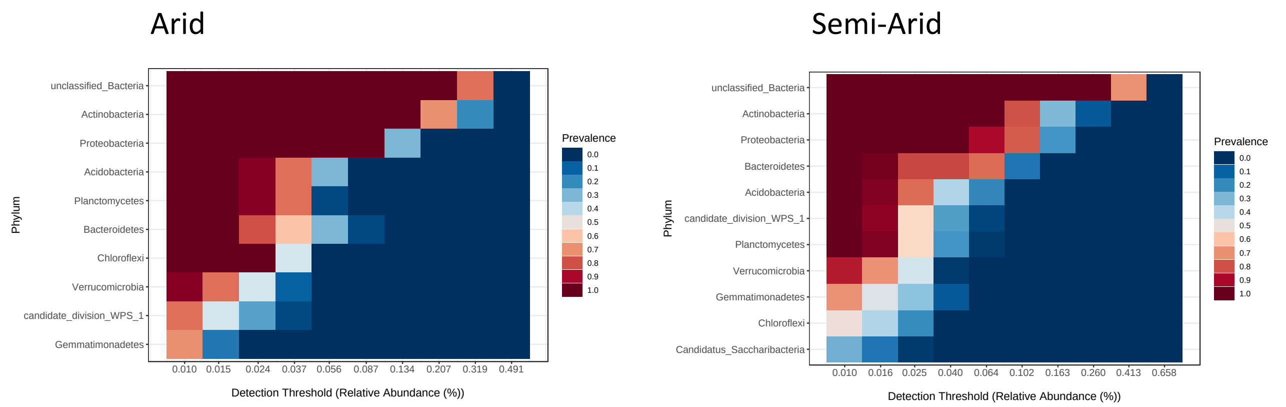

Figure S6.

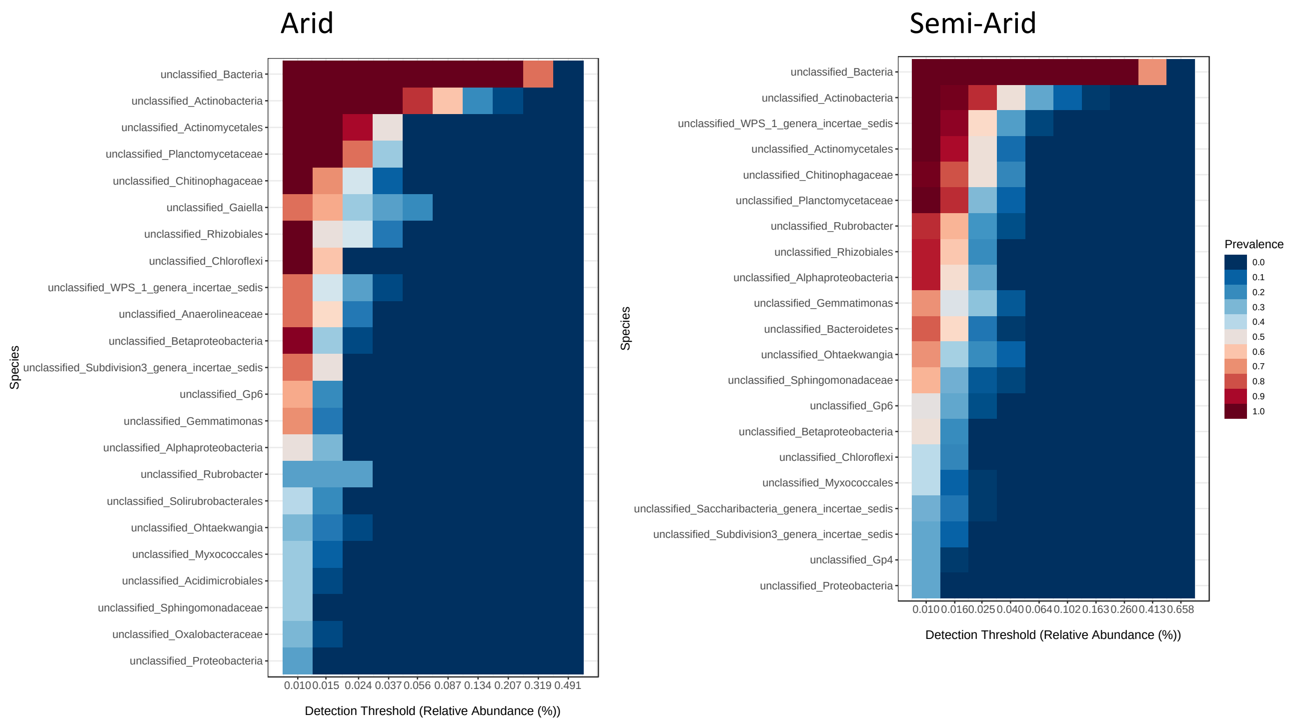

Figure S7.

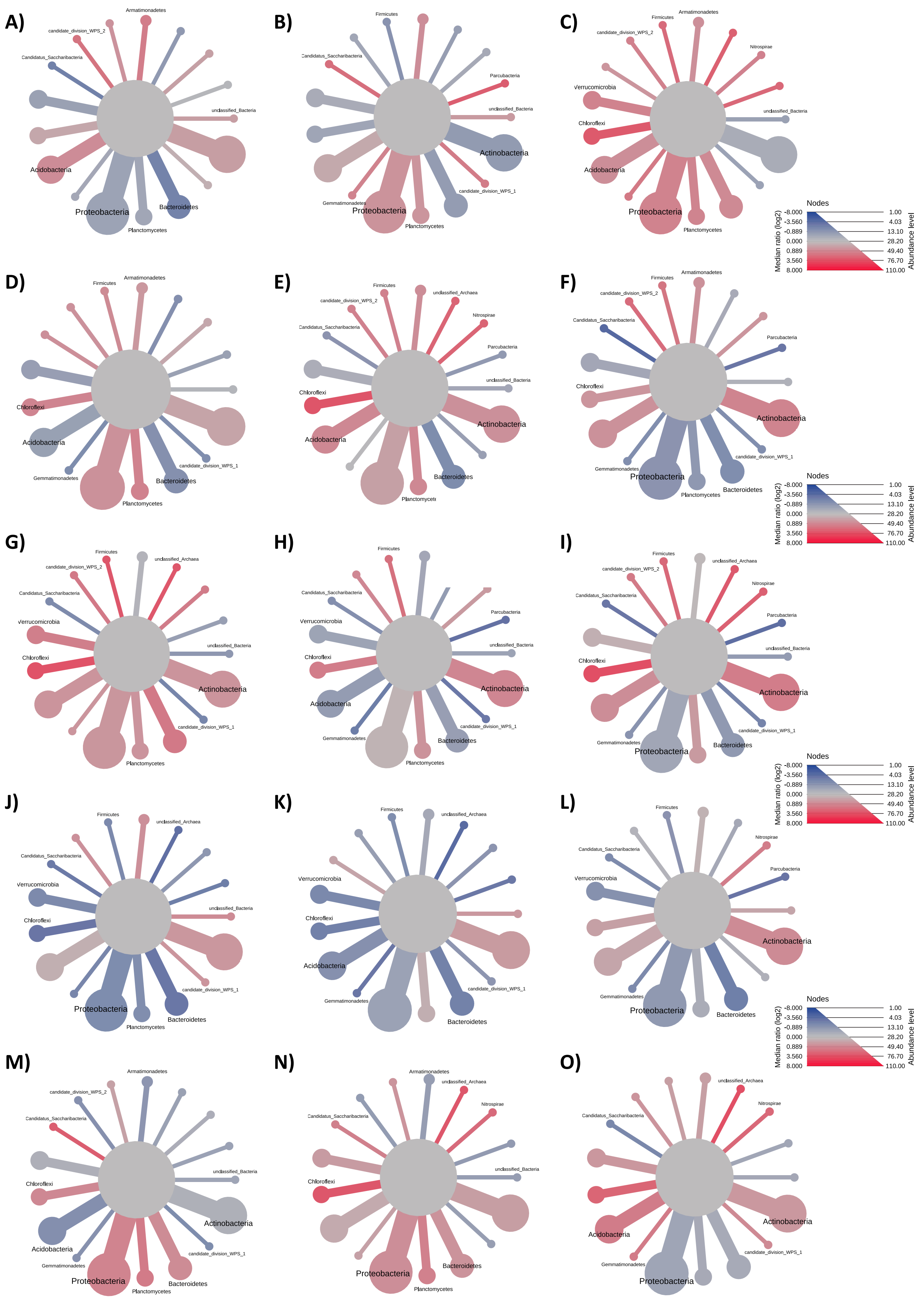
A)
Armatimonadetes
candidate_division_WPS_2
Candidatus_Saccharibacteria
unclassified_Bacteria
Acidobacteria
Proteobacteria
Bacteroidetes
Planctomycetes
B)
Firmicutes
Candidatus_Saccharibacteria
Parcubacteria
unclassified_Bacteria
Actinobacteria
candidate_division_WPS_1
Gemmatimonadetes
Proteobacteria
Planctomycetes
C)
Firmicutes
Armatimonadetes
candidate_division_WPS_2
Nitrospirae
Verrucomicrobia
unclassified_Bacteria
Chloroflexi
Acidobacteria
Proteobacteria
Planctomycetes
Nodes
Median ratio (log2)
Abundance level
-8.000
-3.560
-0.889
0.000
0.889
3.560
8.000
1.00
4.03
13.10
28.20
49.40
76.70
110.00
D)
Firmicutes
Armatimonadetes
Chloroflexi
Acidobacteria
Gemmatimonadetes
candidate_division_WPS_1
Bacteroidetes
Planctomycetes
E)
Firmicutes
unclassified_Archaea
candidate_division_WPS_2
Nitrospirae
Candidatus_Saccharibacteria
Parcubacteria
unclassified_Bacteria
Chloroflexi
Actinobacteria
Acidobacteria
Bacteroidetes
Planctomycete
F)
Firmicutes
Armatimonadetes
candidate_division_WPS_2
Candidatus_Saccharibacteria
Parcubacteria
Chloroflexi
Actinobacteria
candidate_division_WPS_1
Gemmatimonadetes
Proteobacteria
Planctomycetes
Bacteroidetes
G)
Firmicutes
unclassified_Archaea
candidate_division_WPS_2
Candidatus_Saccharibacteria
Verrucomicrobia
unclassified_Bacteria
Chloroflexi
Actinobacteria
candidate_division_WPS_1
Planctomycetes
H)
Firmicutes
Candidatus_Saccharibacteria
Parcubacteria
Verrucomicrobia
unclassified_Bacteria
Chloroflexi
Actinobacteria
Acidobacteria
candidate_division_WPS_1
Gemmatimonadetes
Bacteroidetes
Planctomycetes
I)
Firmicutes
unclassified_Archaea
candidate_division_WPS_2
Nitrospirae
Candidatus_Saccharibacteria
Parcubacteria
unclassified_Bacteria
Chloroflexi
Actinobacteria
candidate_division_WPS_1
Gemmatimonadetes
Bacteroidetes
Proteobacteria
J)
Firmicutes
unclassified_Archaea
Candidatus_Saccharibacteria
Verrucomicrobia
unclassified_Bacteria
Chloroflexi
candidate_division_WPS_1
Proteobacteria
Bacteroidetes
Planctomycetes
K)
Firmicutes
unclassified_Archaea
Verrucomicrobia
Chloroflexi
Acidobacteria
candidate_division_WPS_1
Gemmatimonadetes
Bacteroidetes
L)
Firmicutes
Nitrospirae
Candidatus_Saccharibacteria
Parcubacteria
Verrucomicrobia
Actinobacteria
Gemmatimonadetes
Proteobacteria
Bacteroidetes
M)
Armatimonadetes
candidate_division_WPS_2
Candidatus_Saccharibacteria
unclassified_Bacteria
Chloroflexi
Actinobacteria
Acidobacteria
candidate_division_WPS_1
Gemmatimonadetes
Proteobacteria
Bacteroidetes
Planctomycetes
N)
Armatimonadetes
unclassified_Archaea
Nitrospirae
Candidatus_Saccharibacteria
unclassified_Bacteria
Chloroflexi
Bacteroidetes
Proteobacteria
Planctomycetes
O)
unclassified_Archaea
Nitrospirae
Candidatus_Saccharibacteria
Actinobacteria
Acidobacteria
candidate_division_WPS_1
Gemmatimonadetes
Proteobacteria

Figure S8.
